# Impact of marker density on the accuracy of association mapping

**DOI:** 10.1186/1753-6561-1-s1-s166

**Published:** 2007-12-18

**Authors:** Weihua Zhang, Winston Lau, Cheng Hu, Tai-Yue Kuo

**Affiliations:** 1 Section of Cancer Genetics, The Institute of Cancer Research, 15 Cotswold Road, Belmont, Sutton, Surrey SM2 5NG, UK; 2 Human Genetics Division, Duthie Building (Mailpoint 808), Southampton General Hospital, University of Southampton, School of Medicine, Tremona Road, Southampton, SO16 6YD, UK; 3 Shanghai Diabetes Institute, Shanghai Jiaotong University, 600 Yishan Road, Shanghai 200233, People's Republic of China; 4 Department of Cardiology, Ealing Hospital NHS Trust, Uxbridge Road, Southall, Middlesex, UB1 3HW, UK

## Abstract

We studied the impact of marker density on the accuracy of association mapping using Genetic Analysis Workshop 15 simulated dense single-nucleotide polymorphism (SNP) data on chromosome 6. A total of 1500 cases and 2000 unaffected controls genotyped for 17,820 SNPs were analyzed. We applied the approach that combines information from multiple SNPs under the framework of the Malecot model and composite likelihood to non-overlapping regions of the chromosome. We successfully detected the associations with disease Loci C and D and predicted their locations as small as zero distance to Locus C when it was "typed" and 112 kb from the untyped rare Locus D. Reducing marker density decreased the accuracy of location estimates. However, the predicted locations were robust to variations in the number of SNPs. Generally, the linkage disequilibrium (LD) map reflecting distances between markers in relation to LD produced higher accuracy than the physical map. We also demonstrated that SNP selection based on equal LD distance outperforms that based on equal physical distance or SNP tagging. Furthermore, ignoring rare SNPs diminished the ability to detect rare causal variants.

## Background

As the cost of genotyping decreases, genome-wide association (GWA) mapping of the predisposition genes for complex diseases is becoming a common study design in genetic epidemiology. As the huge number of single-nucleotide polymorphisms (SNPs) in the human genome is still prohibitive for exhaustive investigation, subsets of SNPs have often been selected for large scale studies. Morton et al. developed a novel GWA mapping approach based on the Malecot model and composite likelihood combining multiple marker information from non-overlapping genomic regions to predict the locations of disease variants [1]. We applied this approach to the Genetic Analysis Workshop (GAW) 15 Problem 3 simulated dense chromosome 6 data with the knowledge of the answers and we studied the effect of SNP density on the accuracy of association mapping.

## Methods

### Data

The simulated data set contained 1500 families with a sib pair affected with rheumatoid arthritis (RA) and a random sample of 2000 unrelated and unaffected individuals. To form a case-control study, we selected the first sibling per family as a case. A total of 1500 cases and 2000 controls from Replicate 1 were analyzed. There are three simulated disease loci. HLA-DR is at the same location of 32484.648 kb as Locus C, where a SNP denseSNP6_3437 lies, so we considered this SNP the disease variant C. Locus D is at 37233.784 kb, in very weak linkage disequilibrium (LD) with Locus C. The minor allele frequency (MAF) for the C allele was 0.4055 in control samples. The D allele has a population frequency of 0.0083, but the variant was not typed.

Genotype data were composed of 17,820 SNPs on chromosome 6, mimicking a 300 K GWA scan with no missing values. Fifty-eight SNPs showing departure from Hardy-Weinberg equilibrium (HWE) in control samples (χ^2^_1 _≥ 10 for either Pearson's or likelihood ratio chi-square tests) were discarded [2]. Following convention, 2061 rare SNPs with MAF < 5% were further removed except when otherwise indicated. The main data set (1) was thus composed of a total number of 15,701 SNPs. In another experiment we retained all SNPs but removed 26 SNPs showing departure from HWE by the likelihood-ratio test and this generated 17,794 SNPs (data set 2).

### LD map

The physical map length was 170,813 kb. LD maps expressed in LD units (LDUs) were constructed based on pair-wise LD for multiple markers in control samples [3]. LDU is the product of ε and kb distance for an interval of two adjacent SNPs and is additive, where ε represents the exponential decline of LD with distance for that interval. We used the LDMAP-cluster, a parallel version of LDMAP program that rapidly constructs the maps of equally divided chromosome segments [3]. For each segment, an overall ε value was also estimated. The LD map length was 1311.225 LDUs for the main data set and 1237.923 LDUs for data set 2. SNPs can have the same LDU if they are in an LD block. Therefore, we also made tilted LD maps by reassigning LDU locations for the SNPs with the same LDU by linear interpolation.

### Association mapping

A chromosome is divided into non-overlapping consecutive regions of a minimum number of 30 SNPs and a minimum length of 10 LDUs by default without breaking LD blocks. Each genomic region was then analyzed separately. Association between SNP alleles and disease status in the Malecot model is a function of several parameters. Composite likelihood combines information of all marker-disease association in a genomic region. The parameters were estimated through fitting the model to the data with a map in LDU or kilobases and by minimizing -2 natural log composite likelihood (denoted as Λ) [1]. The estimated location *S *of the disease locus is converted to a kilobase scale. The significance test is performed by contrasting two hierarchical models. Model A assumes no association with the disease, therefore *S *is not estimated. Model D assumes an association with the disease and *S *and two other parameters are estimated and ε is specified. The difference in Λ between models A and D (Λ_A _- Λ_D_) is monotonic to the magnitude of chi-square with three degrees of freedom (χ^2^_3_). Permutation by shuffling case-control status for each region was performed to obtain empirical *p*-values [1]. The algorithms were implemented in the CHROMSCAN program. A parallel version, CHROMSCAN-cluster, deployed on a local Beowulf cluster  was used for computing 1000 replicates.

The values of ε were obtained by averaging over eight segments in LD map construction, which were 1.14472 and 0.00568 for LD and kilobase maps, respectively, for the main data set, and 1.14386 and 0.00544 over nine segments for data set 2. Theoretically, a more accurate ε may be obtained by fitting the maps to the whole chromosome data, but the extensive computing power required for the task is impractical to implement and beyond the current computing resource. Also, slightly altered ε values did not appear to have an appreciable effect (data not shown).

For comparison, a single SNP χ^2^_1 _was obtained by the 2 × 2 allelic count table and the most significant SNP (msSNP) showing maximal χ^2^_1 _in each region was identified. Location error (in kilobases) was defined as the difference between *S *or the location of the msSNP and the true location of disease variant. Accuracy refers to the precision of the predicted location *S*. The smaller the error, the higher the accuracy.

### SNP density

To generate different SNP density, we selected every *i*^th ^SNP (*i *= 2, 3, ..., 20, 25, 30) in the order of their physical locations from the full data set, representing 1/*i *the number of SNPs in the original set. For a candidate region spanning Loci C and D with rare SNPs included, we used Tagger implemented in the Haploview software to select tagging SNPs that optimally capture allelic variation among SNPs at a given *r*^2 ^threshold based on pairwise LD in control samples [4]. For comparison, we selected the same number of SNPs as Tagger but in equal LDU or kilobase distance. To do this we used the tilted LD map in which every SNP had a unique LDU location. We also studied the impact of region length and sample size.

## Results and discussion

### Association mapping of disease loci in full data set

Fourteen out of 126 regions showed nominal significant association with RA (*p *< 0.05), among which eight consecutive regions spanned Loci C and D (Table 1). Five regions remained significant after Bonferroni correction, among which four surrounded or spanned Locus C, and one covered Locus D (Table 1). Locus C was inside the most significant region 29. Therefore, the three regions surrounding Locus C with less significance levels must be the result of LD between variant C and other SNPs. The discontinuity of significance surrounding region 32 indicated that this region harbored another disease locus and indeed, this was where Locus D lies. Therefore, we successfully detected Loci C and D in the initial analysis. The lowest *p *for the rest of the regions was 0.0064. Given that there were no other disease loci, the approach had a right type I error rate (6/118 = 0.05). A lesson learned was that when there was long-range LD, consecutive regions showing association may reflect one instead of several disease loci. As an alternative to merging regions, we studied the impact of region length on accuracy (see below).

**Table 1:** Association mapping of disease Loci C and D on chromosome 6

Region^a^	No. SNPs	*S *(kb)	Λ_A _- Λ_D_	χ^2^_3_	*P*	*P*_c_
26	128	24882	68	14	0.002471	0.311346
27	239	26121	793	40	<10^-7^	0.000001
28	348	31299	5176	128	<10^-12^	<0.000001
29	176	32540	33058	322	<10^-12^	<0.000001
30	153	33962	295	25	0.000017	0.002129
31	134	35638	103	15	0.001929	0.243096
32	127	37776	141	27	0.000007	0.000926
33	147	39432	50	13	0.005554	0.699754

^a^A segment of consecutive regions of 10 LDUs showing nominal significant association with RA (*p *< 0.05). See Methods for the meaning of other symbols. Loci C and D were in regions 29 and 32 at locations of 32485 and 37234 kb, respectively.

^b^P_c _is Bonferroni corrected *p*-value for multiple tests of 126 regions (*p *× 126).

*S *for Locus C was reasonably accurate (55 kb apart from true location using LD map). However, the location error was 542 kb for Locus D and the 95% confidence interval did not include Locus D. Removing 10 SNPs showing significant LD with variant C did not change the results. We then divided region 32 into two or three sub-regions. Again, we did not detect significant association in the middle part where Locus D lies, although we detected the associations in the first and third sub-regions where two clusters of highly significant SNPs lay. Because Locus D is rare, the removal of rare SNPs may have had an effect. We then added rare SNPs and used the corresponding LD map and ε values, and the location accuracy was markedly improved for Locus D (Table 2). Among the added rare SNPs, three were highly associated with the disease: denseSNP6_3931, _3933, and SNP6_162 (χ^2^_1 _= 118, 116, and 116, respectively). It is therefore a mistake to remove rare SNPs (MAF < 0.05) in association analysis. This was in contrast to the HapMap project in which the focus was on common SNPs. However, inclusion of rare SNPs resulted in higher location error for common disease Locus C (Table 2).

**Table 2:** Candidate regions of disease Loci C and D with rare SNPs included

						Location error with rare SNPs	
						
Locus	Map	*S *(kb)	Λ_A _- Λ_D_	χ^2^_3_	*p*	Included	Removed
C	LD	32557	30693	360	<10^-12^	72	55
	LD, tilt	32518	30799	197	<10^-12^	34	21
	kb	32506	28632	496	<10^-12^	22	14
D	LD	37358	154	22	0.000017	124	542
	LD, tilt	37368	156	28	0.000003	130	546
	kb	37368	148	17	0.000666	130	954

Occasionally or under high marker density, the kilobase map performed better than the LD map, presumably because every SNP has a unique physical location, whereas several SNPs could have the same LDU location in LD blocks. The tilted LD map improved the location accuracy for Locus C, although not for Locus D (Table 2).

In practice, the phenomenon in this simulated data set may be too extreme. On the other hand, it is possible that several disease loci can be closely located. To distinguish such loci is a challenge to genetic epidemiologists. Under this circumstance, single SNP association plus a gene functional study may be useful.

### SNP density based on the order

As density decreases, location error increases whether using single or multi-SNP approaches when the disease variant was not "typed" (Table 3). There was an improvement in accuracy when the disease variant was included. In most cases, using the LD map resulted in greater accuracy than using the kilobase map, especially when the marker density was low. We also selected SNPs on the scale of one to the hundredth or even the thousandth. As long as there was one SNP highly associated with the disease (e.g., χ^2^_1 _= 27), the association was detectable, but much compromised by precision as a result of low SNP density. These data are unusual in that the association of Locus C is extremely significant and probably would not be observed in the real data.

**Table 3:** Density and accuracy for Locus C – SNP selection by order

			msSNP		Location error by the composite likelihood approach			
				
					Causal SNP out		Causal SNP in	
						
SNP density (kb/SNP)	No. SNPs	No. regions	χ^2^_1_	Location error	LD	kb	LD	kb
Full (11)	15701	126	2324	153	57	13	55	14
1/2 (22)	7850	125	1762	-2	5	-19	5	-20
1/3 (33)	5233	118	2324	153	153	40	6	40
1/4 (44)	3925	106	1762	-2	-65	-56	-57	-53
1/5 (54)	3140	94	2274	42	-24	-35	-15	-36
1/6 (65)	2616	82	1285	20	20	20	10	15
1/8 (87)	1962	64	1601	65	-58	-64	-47	-58
1/10 (109)	1570	52	726	-106	-55	-59	-24	-46
1/15 (163)	1046	34	486	-887	294	26	0	3
1/20 (217)	785	26	726	-106	-97	-160	-26	-43
1/25 (272)	628	20	348	-9	69	-120	60	-79
1/30 (326)	523	17	229	188	362	-25	0	-25

Disease variant C (χ^2^_1 _= 1916) was not present except in the full data set or specified.

Although mapping accuracy decreases with marker density, even with 1/30 the number of SNPs, corresponding to a 10 K GWA scan, we could still detect Locus C (Table 3). Single SNP tests depend heavily on whether the disease variant is typed. It has less predictive value for accuracy because the SNP with maximal χ^2 ^is not necessarily the closest SNP to the disease variant. In contrast, methods that combine information from multiple markers predict the location of the disease variant better than single SNP tests because the location is less influenced by any single SNP effects. A multi-marker approach may therefore be more robust to genotyping errors.

We expect that the mapping accuracy will be improved further in maps with higher marker density than that assessed in this paper, such as the commercially available 500 K or more genotyping platforms for GWA studies.

### SNP density based on tagging or equidistance

For the 15,805.710 kb candidate region spanning both Loci C and D, we compared location accuracy using SNPs selected with Tagger or by equidistance of LDU or kilobases (Table 4). SNPs based on equal LDU provided higher location accuracy than those based on equal kilobase distance. Equidistance generally provided higher accuracy than tagging SNP selection. Again, reducing SNP density decreases the prediction accuracy of disease Loci C and D, but this was minimally affected by selection based on equal LD distance (Table 4).

**Table 4:** Density and accuracy – SNP selection by tagging or equidistance^a^

		Locus C			Locus D		
			
*R*^2 ^(LDU, kb)	No. SNPs (kb/SNP)	Tagger	E_LD	E_kb	Tagger	E_LD	E_kb
Full	1658 (10)	20	20	20	130	130	130
0.8 (0.013,7)	1080^b ^(15)	35	20	30	130	124	130
0.6 (0.025,10)	874 (18)	56	-16	27	545	124	551
0.4 (0.047,15)	657 (24)	106	42	63	124	123	117
0.2 (0.099,27)	421 (38)	125	14	-23	537	112	231

^a^Location error for SNPs selected by Tagger or equal LDU (E_LD) or kb (E_kb) distance in a candidate region of 15805.710 kb with rare SNPs included. Regions were fixed at 10 LDUs for Loci C (30997–33398 kb) and D (36784–37792 kb). Disease variant C was not present except in the full data set. Tilted LD map.

^b^1079 for E_LD.

### Sample size and region length

We analyzed different sample sizes based on the combination of 500, 1000, 1500, and 2000 cases or controls. Despite variations in location errors for Locus C, there was no clear trend to draw any meaningful conclusion. For Locus D, however, a high degree of accuracy appeared to be maintained when the data sets had over 1000 cases and 1500 controls. Therefore, large samples are needed for detecting rare disease loci.

With Locus C being centred, we studied region lengths from 0.2 up to 30 LDUs, with the latter starting in region 27 and ending in region 30. The location error was relatively stable but extremely small or large LDU lengths resulted in increased error. The region lengths in LDUs (location errors in kilobases) were 0.2 (107), 1 (5), 2 (82), 4 (5), 6 (5), 8 (5), 10 (5), 12 (-10), 14 (-10), 16 (-13), 18 (-14), 20 (-14), and 30 (-68). We therefore recommend 10-LDU for the maximal length while maintaining minimal error. Increasing the number of SNPs also linearly increases the computing load [3].

Fixing region length had no appreciable impact on location accuracy at high density, but the errors were greater than let-the-program-decide regions at low density (data not shown).

## Conclusion

We successfully detected disease Loci C and D in the simulated dense chromosome 6 data using the Malecot model and composite likelihood approach. Decreasing SNP density compromises accuracy of association mapping. This multi-marker approach has many advantages. Firstly, it markedly decreases the number of tests in GWA studies, avoiding heavy penalty for multiple testing. Secondly, it predicts the disease loci more accurately than single SNP association tests. We also demonstrated that SNP selection by equal LD distance outperforms that by tagging or equal kilobase distance in the accuracy of association mapping. Finally, we conclude that excluding rare SNPs significantly decreases the power and accuracy in mapping rare disease loci.

## Competing interests

The author(s) declare that they have no competing interests.
